# Permittivity evaluation of electrically thin low-loss materials by using a generalized free-space model without any formal calibration

**DOI:** 10.1038/s41598-026-50597-w

**Published:** 2026-05-23

**Authors:** Ugur C. Hasar, Huseyin Korkmaz, Yunus Kaya

**Affiliations:** 1 Department of Electrical and Electronics Engineering, Gaziantep University, Gaziantep, 27310 Türkiye; 2 Applied Electromagnetics Research Laboratory (Aer-Lab), Gaziantep University, Gaziantep, 27310 Türkiye; 3 Department of Electrical and Electronics Engineering, Kilis 7 Aralık University, Kilis, 79000 Türkiye; 4 Department of Electrical and Electronics Engineering, Ataturk University, Erzurum, 25240 Türkiye

**Keywords:** Engineering, Physics

## Abstract

A general free-space model based on reflection and transmission measurements is proposed for accurate permittivity determination of low-loss and electrically thin (*W < λ /4* where *W* is the sample thickness and *λ* is the wavelength) samples. Unlike previous free-space methods studied in the literature, the proposed new model generalizes previous free-space models by considering the case that transmitting and receiving antenna regions have different reflection and transmission properties. Such a model, valid at any frequency or over the entire frequency band, considers different antenna characteristics or different cable connections in addition to unequal and non-zero reflection properties due to impedance mismatches and ringing effects between antennas and the sample. An objective function constructed on this model is derived. This function is shown to be free-from error network terms of transmitting and receiving antenna regions and not dependent on reference-plane transformation factors. The effect of sample misalignment is considered to evaluate its effect on permittivity determination by the proposed extraction method. Free-space measurements at X-band (8.2–12.4 GHz) of low-loss Rogers 4350B sample (*W ≈ 1.52* mm) and FR-4 sample (*W ≈ 1.60* mm) were then carried out to validate our method.

## Introduction

Microwave electromagnetic characterization constitutes one of the building blocks of microwave nondestructive testing and evaluation^1^. Such a characterization can be realized by non-resonance based methods thanks to their broadband nature, relatively relaxed sample preparation requirement, and widespread implementability over many distinct application areas involving composition analysis and cure state monitoring of cement-based structures, detection of conductive filler in rubber composites, and examining porosity level in ceramics^2,3^. Among many non-resonance based methods (planar, coaxial, waveguide, free-space, etc.), free-space non-resonance based methods have distinct advantages involving simplicity of analysis in frequency-domain^4–22^ or time-domain^23–27^ and non-contact measurements, especially for measurements at harsh environmental conditions^6^.

There are four main scenarios considered in the analysis of free-space methods: i) implementation using reflection-only^15,18^, transmission-only^12,17^, or reflection and transmission measurements^8,13^, ii) dependence on partially-calibrated or self-calibrated (without determining elements of any error networks) measurements^12,14,16,18^, iii) extraction relying on analytical expressions without utilizing any numerical tool or vice versa^8,11^, and iv) electromagnetic parameter retrieval of thin materials^19^. It is noted that while reflection-only free-space measurements provide sufficient information for material characterization and are suitable for measurements of samples whose rear side is not achievable^17,20,22^, reflection and transmission free-space measurements give additional information for the same goal^19^ and are feasible for through-wall applications^28^. Free-space methods requiring sample thickness information are highly dependent on sample thickness information for an accurate electromagnetic parameter determination^21^, especially for thinner samples due to fabrication tolerances. In addition, free-space methods based on any formal calibration procedure^4–13,15,17,19,22^ are limited by a proper calibration procedure based on calibration standards. To eliminate such a dependence, self-calibrated or partial-calibrated free-space methods relying on self-calibration procedures^14,16,18–21^ can be exercised. Besides, free-space methods utilizing a numerical toolbox suffer from improper initial guess for electromagnetic properties of the sample. Finally, electromagnetic property measurement of thin materials is also important due to its importance in many diverse fields including microwave absorbers^29^. In the present study, our goal is to propose a free-space electromagnetic extraction procedure for electrically thin samples relying on an improved self-calibrated procedure using reflection and transmission measurements without resorting to any numerical toolbox.

When the free-space methods^14,16,18–21^ using self-calibrated or partial-calibrated are examined, it is noted that only those in the studies^16,19,21^ utilize both reflection and transmission free-space measurements, which are our concern in this study. The free-space extraction methods^16,19,21^ use two two-port networks to take into account of transmitting and receiving antenna regions. These models in the studies^16,21^ assume identical two two-port networks. However, such an assumption is hard to fully realize in real applications due to some fabrication tolerances that may appear even if transmitting and receiving antennas are fabricated from the same batch. The error network model in the study^19^ can be utilized to relax this stringent requirement. Nonetheless, this method considers that these networks have zero internal reflection. Such a consideration can result in reduced accuracy for antennas having large internal reflections, which then produce ripples in the extracted $\varepsilon _r$ due to ringing between the sample and antenna. Table 1 presents a brief comparison of our proposed method with other reflection-only free-space methods^14,18,20^ and reflection-transmission free-space methods^16,19,21^ using calibration error networks in the extraction procedure in terms of measurement type, properties of error networks, whether error networks involve zero or non-zero reflection, and whether error networks are reciprocal or non-reciprocal.

In this paper, dissimilar from the studies^16,19,21^, transmitting and receiving antenna region error networks are considered to be non-reciprocal and non-identical with zero or non-zero reflection asymmetric feature to improve $\varepsilon _r$ determination. A metric function with no dependence on the terms of the transmitting and receiving antenna networks and with no reference plane transformation information is determined for accurate and stable $\varepsilon _r$ extraction. The remaining part of our paper is formatted as follows. First, the proposed method along with derived objective functions for $\varepsilon _r$ determination is presented in “The proposed method”. Next, results of a numerical analysis for validation of our method are analyzed in “Validation”. Then, analysis of angle misalignment effect is followed in “Effect of sample misalignment”. After, discussion of measurement results of two low-loss and electrically thin samples is given in “Measurement results and discussion”, and pros and cons of our method are mentioned in “Advantages and limitations of our method”. Finally, all relevant points are recapitulated in “Conclusion”.

**Table 1:** Comparison of some free-space reflection/transmission extraction methods.

Study	Measurement type	Error network for antennas	Reflection property of error networks	Reciprocal or non-reciprocal
^14^	Reflection	One Two-Port Network	Negligible or Zero-Reflection	Reciprocal
^18^	Reflection	One Two-Port Network	Negligible or Zero-Reflection	Nonreciprocal
^20^	Reflection	One Two-Port Network	Negligible or Zero-Reflection	Reciprocal
^16^	Reflection/Transmission	Identical Two Two-Port Networks	Zero-Reflection	Reciprocal
^19^	Reflection/Transmission	Non-Identical Two Two-Port Networks	Zero-Reflection	Reciprocal
^21^	Reflection/Transmission	Identical Two Two-Port Networks	Non-Zero Reflection	Reciprocal
Proposed	Reflection/Transmission	Non-Identical Two Two-Port Networks	Non-Zero Reflection	Nonreciprocal

## The proposed method

### Description of error matrices and sample region

**Figure 1:**
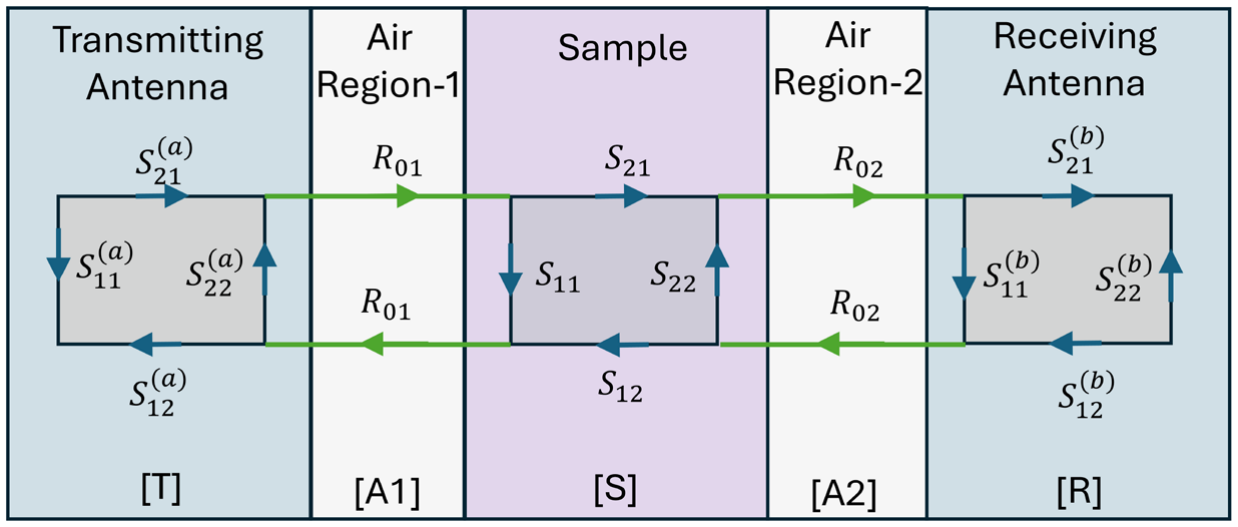
A schematic description of a free-space measurement environment involving three full two-port networks (transmitting antenna region [*T*], receiving antenna region [*R*], and the sample region [*S*]) and two simplified two-port networks (reflection-free air regions [*A*1], and [*A*2]).

Figure 1 illustrates a schematic description of a free-space measurement environment involving five distinct regions, three of which are described by full two-port networks (transmitting and receiving antenna regions in addition to the sample region) and two of which are described by simplified two-port networks (reflection-free air regions). While $S_{11}^{\text {(a)}}$ and $S_{21}^{\text {(a)}}$ are the forward scattering (S-) parameters and $S_{12}^{\text {(a)}}$ and $S_{22}^{\text {(a)}}$) are the backward S-parameters of the transmitting antenna region [*T*], $S_{11}^{\text {(b)}}$ and $S_{21}^{\text {(b)}}$ are the forward S-parameters and $S_{12}^{\text {(b)}}$ and $S_{22}^{\text {(b)}}$ are the backward S-parameters of the receiving antenna region [*R*]. In a similar fashion, $S_{11}$, $S_{21}$, $S_{12}$, and $S_{22}$ are the forward and backward reflection and transmission S-parameters of the sample [*S*]. On the other hand, $R_{01}$ and $R_{02}$ are the (forward or backward) transmission S-parameters of the air regions [*A*1] and [*A*2], respectively. It is noted that the free-space model considered here is more general than that considered in the study^16^, which assumes $S_{11}^{\text {(a)}} = S_{22}^{\text {(a)}} = S_{11}^{\text {(b)}} = S_{22}^{\text {(b)}} = 0$ and $S_{21}^{\text {(a)}} = S_{12}^{\text {(a)}} = S_{21}^{\text {(b)}} = S_{12}^{\text {(b)}}$ and that examined in the study^19^, which uses $S_{11}^{\text {(a)}} = S_{22}^{\text {(a)}} = S_{11}^{\text {(b)}} = S_{22}^{\text {(b)}} = 0$, $S_{21}^{\text {(a)}} = S_{12}^{\text {(a)}} = T_a$, and $S_{21}^{\text {(b)}} = S_{12}^{\text {(b)}} = T_b$, and that formulated in the study^21^, which utilizes $S_{11}^{\text {(a)}} = S_{22}^{\text {(b)}}$, $S_{11}^{\text {(b)}} = S_{22}^{\text {(a)}}$, $S_{12}^{\text {(a)}} = S_{21}^{\text {(a)}}$, and $S_{12}^{\text {(b)}} = S_{21}^{\text {(b)}}$.

**Figure 2:**
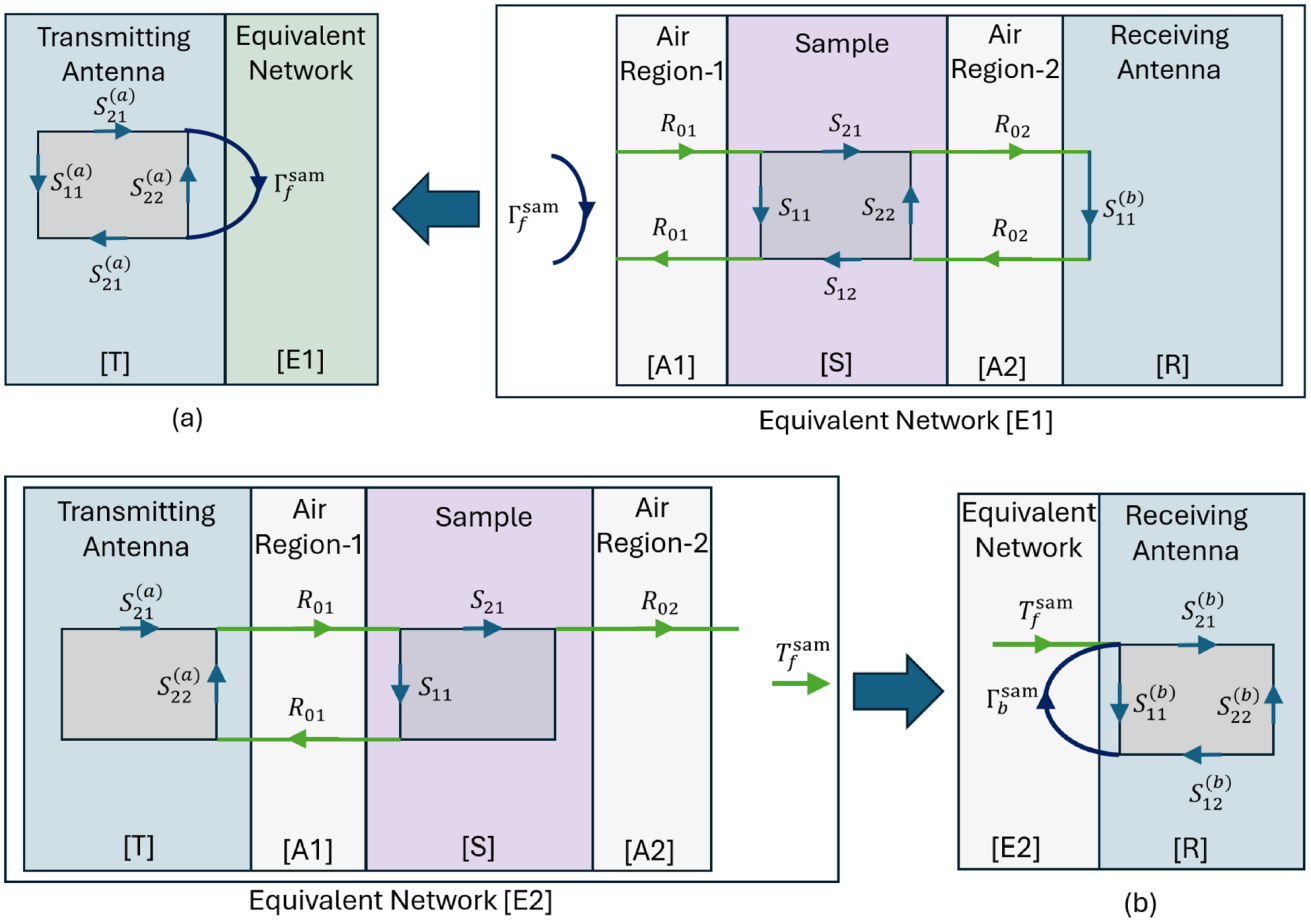
Sub-models for the analysis of forward (**a**) reflection and (**b**) transmission S-parameters of the measurement system. Here, $\Gamma _f^{\text {sam}}$ and $\Gamma _b^{\text {sam}}$ are the equivalent reflection S-parameters while $T_f^{\text {sam}}$ is the equivalent transmission S-parameter.

For the cascaded network description in Fig. 1, it is possible to apply wave-cascading matrices, transfer matrices, or state-transition matrices to examine overall reflection and transmission properties of the entire system. However, we will prefer using S-parameter description of this cascaded network description, although partly complicates the analysis, because determinant of a two-port S-parameter description is free from the effects of rotations (reference-plane-invariant property)^30^. The overall system in Fig. 1 can be evaluated using the sub-models in Fig. 2a,b for forward reflection and transmission S-parameters, respectively. In these sub-models, $\Gamma _f^{\text {sam}}$ and $\Gamma _b^{\text {sam}}$ refer to the equivalent reflection S-parameters seen at the forward and backward directions whereas $T_f^{\text {sam}}$ presents the equivalent transmission S-parameter seen at the forward direction^31^.

Utilizing the sub-models in Fig. 2a,b, one can determine the forward reflection and transmission S-parameters of the entire system ($S_{11}^{\text {sam}}$ and $S_{21}^{\text {sam}}$) as

1 $$\begin{aligned} S_{11}^{\text {sam}} = S_{11}^{\text {(a)}} + \frac{ S_{21}^{\text {(a)}} S_{12}^{\text {(a)}} \Gamma _f^{\text {sam}}}{ 1 - \Gamma _f^{\text {sam}} S_{22}^{\text {(a)}} }, \end{aligned}$$

2 $$\begin{aligned} S_{21}^{\text {sam}} = \frac{ S_{21}^{\text {(b)}} T_f^{\text {sam}} }{ 1 - \Gamma _b^{\text {sam}} S_{11}^{\text {(b)}} }, \end{aligned}$$

where

3 $$\begin{aligned} \Gamma _f^{\text {sam}} = R_{01}^2 \left( S_{11} + \frac{ R_{02}^2 S_{21} S_{12} S_{11}^{\text {(b)}} }{ 1 - R_{02}^2 S_{22} S_{11}^{\text {(b)}} } \right) , \end{aligned}$$

4 $$\begin{aligned} \Gamma _b^{\text {sam}} = R_{02}^2 \left( S_{22} + \frac{ R_{01}^2 S_{12} S_{21} S_{22}^{\text {(a)}} }{ 1 - R_{01}^2 S_{11} S_{22}^{\text {(a)}} } \right) , \end{aligned}$$

5 $$\begin{aligned} T_f^{\text {sam}} = R_{01} R_{02} \frac{ S_{21} S_{21}^{\text {(a)}} }{ 1 - R_{01}^2 S_{11} S_{22}^{\text {(a)}} }. \end{aligned}$$

Following a similar procedure, the backward reflection and transmission S-parameters of the entire system ($S_{22}^{\text {sam}}$ and $S_{12}^{\text {sam}}$, respectively) can be obtained as

6 $$\begin{aligned} S_{22}^{\text {sam}} = S_{22}^{\text {(b)}} + \frac{ S_{12}^{\text {(b)}} S_{21}^{\text {(b)}} \Gamma _b^{\text {sam}}}{ 1 - \Gamma _b^{\text {sam}} S_{11}^{\text {(b)}} }, \end{aligned}$$

7 $$\begin{aligned} S_{12}^{\text {sam}} = \frac{ S_{12}^{\text {(a)}} T_b^{\text {sam}} }{ 1 - \Gamma _f^{\text {sam}} S_{22}^{\text {(a)}} }, \end{aligned}$$

where

8 $$\begin{aligned} T_b^{\text {sam}} = R_{01} R_{02} \frac{ S_{12} S_{12}^{\text {(b)}} }{ 1 - R_{02}^2 S_{22} S_{11}^{\text {(b)}} }. \end{aligned}$$

It is noted that symmetry exits between forward and backward reflection (and transmission) S-parameters in (1)-(8). For example, $S_{12}^{\text {sam}}$ can be evaluated from (2) by proper interchange of parameters.

For the case after the sample is removed from the arrangement in Fig. 1 (air-filled region), forward and backward S-parameters of this arrangement ($S_{11}^{\text {air}}$, $S_{21}^{\text {air}}$, $S_{12}^{\text {air}}$, and $S_{22}^{\text {air}}$) will have the following expressions

9 $$\begin{aligned} S_{11}^{\text {air}} = S_{11}^{\text {(a)}} + \frac{ S_{21}^{\text {(a)}} S_{12}^{\text {(a)}} \Gamma _f^{\text {air}}}{ 1 - \Gamma _f^{\text {air}} S_{22}^{\text {(a)}} }, \end{aligned}$$

10 $$\begin{aligned} S_{21}^{\text {air}} = \frac{ S_{21}^{\text {(b)}} T_f^{\text {air}} }{ 1 - \Gamma _b^{\text {air}} S_{11}^{\text {(b)}} }, \end{aligned}$$

11 $$\begin{aligned} S_{22}^{\text {air}} = S_{22}^{\text {(b)}} + \frac{ S_{12}^{\text {(b)}} S_{21}^{\text {(b)}} \Gamma _b^{\text {air}}}{ 1 - \Gamma _b^{\text {air}} S_{11}^{\text {(b)}} }, \end{aligned}$$

12 $$\begin{aligned} S_{12}^{\text {air}} = \frac{ S_{12}^{\text {(a)}} T_b^{\text {air}} }{ 1 - \Gamma _f^{\text {air}} S_{22}^{\text {(a)}} }, \end{aligned}$$

where

13 $$\begin{aligned} \Gamma _f^{\text {air}} = R_{01}^2 R_{02}^2 S_{21}^0 S_{12}^0 S_{11}^{\text {(b)}}, \end{aligned}$$

14 $$\begin{aligned} \Gamma _b^{\text {air}} = R_{02}^2 R_{01}^2 S_{12}^0 S_{21}^0 S_{22}^{\text {(a)}}, \end{aligned}$$

15 $$\begin{aligned} T_f^{\text {air}} = R_{01} R_{02} S_{21}^0 S_{21}^{\text {(a)}}, \end{aligned}$$

16 $$\begin{aligned} T_b^{\text {air}} = R_{01} R_{02} S_{12}^0 S_{12}^{\text {(b)}}. \end{aligned}$$

It is noteworthy that $S_{11} = S_{22} = 0$, $S_{21} \rightarrow S_{21}^0$, and $S_{12} \rightarrow S_{12}^0$ for this new arrangement.

### Determination of relative complex permittivity free from error networks and reference plane transformation factors

Given the expressions of $S_{11}^{\text {sam}}$, $S_{21}^{\text {sam}}$, $S_{12}^{\text {sam}}$, and $S_{22}^{\text {sam}}$ in (1), (2), (6), and (7) and the expressions of $S_{11}^{\text {air}}$, $S_{21}^{\text {air}}$, $S_{12}^{\text {air}}$, and $S_{22}^{\text {air}}$ in (9)-(12), the determinant of S-parameters corresponding to the sample and air cases can be obtained as

17 $$\begin{aligned}&S_{11}^{\text {u}} S_{22}^{\text {u}} - S_{21}^{\text {u}} S_{12}^{\text {u}} = \Delta S_{\text {u}} = \nonumber \\&\dfrac{ S_{11}^{\text {(a)}} S_{22}^{\text {(b)}} - S_{21}^{(b)} S_{12}^{(a)} T_f^{\text {u}} T_b^{\text {u}} - \Delta S_a S_{22}^{\text {(b)}} \Gamma _f^{\text {u}} + \Delta S_b \Gamma _b^{\text {u}} \left( \Delta S_a \Gamma _f^{\text {u}} - S_{11}^{\text {(a)}} \right) }{ \left[ 1 - \Gamma _b^{\text {u}} S_{11}^{\text {(b)}} \right] \left[ 1 - \Gamma _f^{\text {u}} S_{22}^{\text {(a)}} \right] }, \end{aligned}$$

where u = ‘sam’ or ‘air’ and

18 $$\begin{aligned} \Delta S_a = S_{11}^{\text {(a)}} S_{22}^{\text {(a)}} - S_{12}^{\text {(a)}} S_{21}^{\text {(a)}}, \ \ \ \ \ \ \Delta S_b = S_{11}^{\text {(b)}} S_{22}^{\text {(b)}} - S_{12}^{\text {(b)}} S_{21}^{\text {(b)}}. \end{aligned}$$

Similarly, hybrid products of reflection S-parameters of the sample and air regions yield

19 $$\begin{aligned}&S_{11}^{\text {sam}} S_{22}^{\text {air}} - S_{11}^{\text {air}} S_{22}^{\text {sam}} = S_{\text {mix}} \nonumber \\&= \left[ \frac{ S_{11}^{\text {(a)}} - \Delta S_a \Gamma _f^{\text {sam}} }{ 1 - \Gamma _f^{\text {sam}} S_{22}^{\text {(a)}} } \right] \left[ \frac{ S_{22}^{\text {(b)}} - \Delta S_b \Gamma _b^{\text {air}} }{ 1 - \Gamma _b^{\text {air}} S_{11}^{\text {(b)}} } \right] \nonumber \\&+ \left[ \frac{ S_{11}^{\text {(a)}} - \Delta S_a \Gamma _f^{\text {air}} }{ 1 - \Gamma _f^{\text {air}} S_{22}^{\text {(a)}} } \right] \left[ \frac{ S_{22}^{\text {(b)}} - \Delta S_b \Gamma _b^{\text {sam}} }{ 1 - \Gamma _b^{\text {sam}} S_{11}^{\text {(b)}} } \right] . \end{aligned}$$

It is clearly seen from (17)-(19) that $\Delta S_{\text {sam}}$, $\Delta S_{\text {air}}$, and $S_{\text {mix}}$ vary with $\Delta S_a$ and $\Delta S_b$. If the sample has reflection symmetric property and is reciprocal, one can evaluate^21^

20 $$\begin{aligned}&S_{11} = S_{22} = \frac{ \Gamma (1 - T^2) }{ 1 - \Gamma ^2 T^2 }, \ \ S_{21} = S_{12} = \frac{T (1 - \Gamma ^2) }{1 - \Gamma ^2 T^2}, \end{aligned}$$

21 $$\begin{aligned} \Gamma = \frac{1 - \sqrt{\varepsilon _r} }{1 + \sqrt{\varepsilon _r} }, \ \ \ T = e^{- j k_0 \sqrt{\varepsilon _r} W }, \ \ \ \varepsilon _r = \varepsilon _r^{\prime } - j \varepsilon _r^{\prime \prime }, \end{aligned}$$

where the surface reflection coefficient and propagation factor in the sample are denoted by *Γ* and *T*, respectively, and the sample thickness and free-space wavenumber are represented by *W* and $k_0$, respectively.

On the other hand, the transmission S-parameters for the air region [A1] with length $L_{01}$, the air region [A2] with length $L_{02}$, and the air region with length *W* (no sample is present) in Fig. 1 can be evaluated from

22 $$\begin{aligned} R_{01} = e^{- j k_0 L_{01} }, \ \ \ R_{02} = e^{- j k_0 L_{02} }, \ \ \ T_0 = e^{- j k_0 W }. \end{aligned}$$

It is obvious from (17) and (19) that $\Delta S_{\text {sam}}$, $\Delta S_{\text {air}}$, and $S_{\text {mix}}$ are not only all functions of error network terms ($S_{11}^{\text {(a)}}$, $S_{21}^{\text {(a)}}$, $S_{212}^{\text {(a)}}$, $S_{22}^{\text {(a)}}$) and but also are dependent on reference plane transformation factors $R_{01}$ and $R_{02}$. Similar to the study^21^, the following objective function can be ascertained after utilizing $\Delta S_{\text {sam}}$, $\Delta S_{\text {air}}$, and $S_{\text {mix}}$ in (17)-(19)

23 $$\begin{aligned}&F_{\text {obj}} = \frac{ S_{\text {mix}} - \Delta S_{\text {sam}} - \Delta S_{\text {air}} }{ S_{21}^{\text {sam}} S_{21}^{\text {air}} } = \frac{ T_0^2 (1 - \Gamma ^2 T^2 ) + (T^2 - T_0^2) }{ T_0 T (1 - \Gamma ^2)} \nonumber \\&= \bigg [ 1 + \frac{1}{2} \left( \sqrt{\varepsilon _r} + \frac{1}{\sqrt{\varepsilon _r}} \right) \bigg ] \text {cos} \left[ (1 - \sqrt{\varepsilon _r}) k_0 W \right] \nonumber \\&+ \bigg [ 1 - \frac{1}{2} \left( \sqrt{\varepsilon _r} + \frac{1}{\sqrt{\varepsilon _r}} \right) \bigg ] \text {cos} \left[ (1 + \sqrt{\varepsilon _r}) k_0 W \right] \end{aligned}$$

It is clear from the expression of $F_{\text {obj}}$ in (23) that $\varepsilon _r$ can be directly evaluated from measured S-parameters without resorting to the information of the error network terms and reference plane transformation factors. Such an evaluation, however, requires a 2D numerical methodology such as the Newton-Raphson method. There are two drawbacks of such a computation. First, numerical methods necessitate a good initial guess to converge to a global minimum. Second, 2D numerical methods require more computational time than 1D numerical methods necessitate (e.g., internal halving or bisection method)^32^. After considering these points, letting $\sqrt{\varepsilon _r} = a - j b$ and $\phi _0 = k_0 W$, and assuming $\text {sinh} (b \phi _0) \approx b \phi _0$ and $\text {cosh} (b \phi _0) \approx 1.0$ for electrically thinner samples (*W < λ /4* where *λ* is the sample wavelength), the following useful objective function is derived to evaluate *a* only

24 $$\begin{aligned}&G_{\text {obj}} (a) \approx 0 = \nonumber \\&\Bigg | F_{\text {obj}}^{\text {real}} - \left( a + \frac{1}{a} \right) \text {sin} (a \phi _0) \text {sin} (\phi _0) + 2 \text {cos} (a \phi _0) \text {cos} (\phi _0) \Bigg |, \end{aligned}$$

where $F_{\text {obj}}^{\text {real}}$ is the real part of $F_{\text {obj}}$, and *|⋆ |* denotes the magnitude of ‘*⋆*’. Even though the objective function given in (24) is identical to that in the study^21^, our model in Fig. 1 is more general than that was considered in the study^21^. For example, the new model examined in Fig. 1 considers that there is no need that the transmitting and receiving antennas are identical having the same reflection and transmission properties $S_{11}^{\text {(a)}} = S_{22}^{\text {(b)}}$, $S_{22}^{\text {(a)}} = S_{11}^{\text {(b)}}$, $S_{21}^{\text {(b)}} = S_{12}^{\text {(b)}}$, and $S_{12}^{\text {(a)}} = S_{21}^{\text {(b)}}$.

After substituting the calculated *a* from (24), *b* can be computed by a suitable 1D numerical method from

25 $$\begin{aligned}&H_{\text {obj}} (b) \approx 0 = \nonumber \\&\Bigg | F_{\text {obj}}^{\text {real}} - \Bigg [ \left( \frac{b}{a^2 + b^2} - b \right) \text {cos} (a \phi _0) \text {sinh} (b \phi _0) \nonumber \\&+ \left( \frac{a}{a^2 + b^2} + a \right) \text {sin} (a \phi _0) \text {cosh} (b \phi _0) \Bigg ] \text {sin} (\phi _0) \nonumber \\&+ 2 \text {cos} (\alpha \phi _0) \text {cos} (\phi _0) \text {cosh} (b \phi _0) \Bigg |. \end{aligned}$$

Finally, $\varepsilon _r$ can be determined from

26 $$\begin{aligned} \varepsilon _r^{\prime } = a^2 - b^2, \ \ \ \varepsilon _r^{\prime \prime } = 2 a b. \end{aligned}$$

It is noted that the derived objective functions in (24) and (25) are applicable for electrically thin low-loss samples, which are our main concern in the present study.

**Figure 3:**
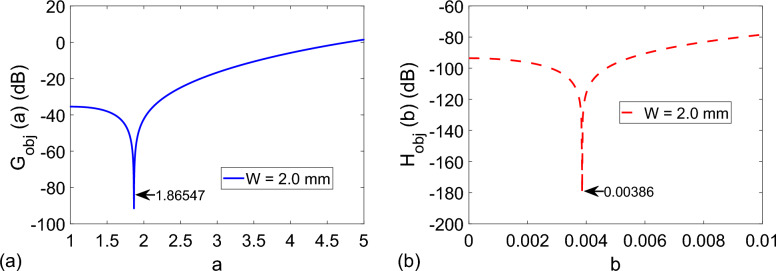
Dependencies of (**a**) $G_{\text {obj}} (a)$ versus *a* and (**b**) $H_{\text {obj}} (b)$ versus *b* of the tested sample (*W = 2.00* mm).

**Figure 4:**
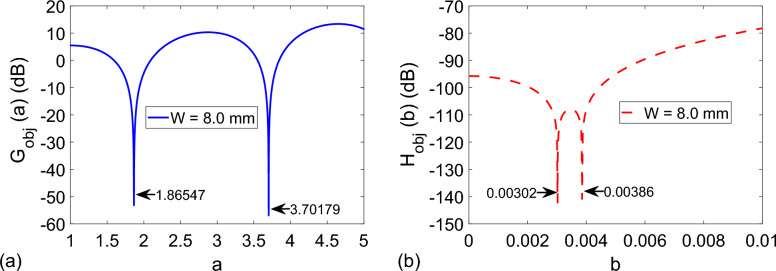
Dependencies of (**a**) $G_{\text {obj}} (a)$ versus *a* and (**b**) $H_{\text {obj}} (b)$ versus *b* of the tested sample when *W = 8.00* mm.

## Validation

In this section, a numerical analysis is considered to validate the derived objection functions $G_{\text {obj}} (a)$ in (24) and $H_{\text {obj}} (b)$ in (25). Toward this end, the parameters, selected arbitrarily just for the demonstration purpose, were considered: $S_{11}^{(a)} = 0.1 - j 0.4$, $S_{21}^{(a)} = S_{12}^{(a)} = 0.5 + j 0.4$, $S_{22}^{(a)} = -0.2 - j 0.2$, $S_{11}^{(b)} = 0.1 + j 0.2$, $S_{21}^{(b)} = S_{12}^{(b)} = -0.2 + j 0.4$, and $S_{22}^{(b)} = -0.7 - j 0.1$, $L_{01} = 40$ cm, and $L_{02} = 42$ cm ($L_{01} = L_{02}$ could also be used). In addition, it was assumed that the sample had $\varepsilon _r = 3.48(1 - j0.0037)$ (Rogers 4350B)^21,33^, *W = 2.0* mm, and *f = 10.3* GHz (mid-frequency) after taking into account of the measurement configuration given in “Measurement results and discussion”. For this selection, it is noted that $\lambda \approx 15.6$ mm and $W \ll \lambda /4$. Figure 3a,b demonstrate, respectively, the dependencies of $G_{\text {obj}} (a)$ versus *a* and $H_{\text {obj}} (b)$ versus *b* for the tested sample. It is seen from Fig. 3a,b that there is only one *a* value and one *b* value, which are very close to the actual values of *≈ 1.86547* and *≈ 0.00386* [$\varepsilon _r \cong 3.479 (1 - j 0.0040)$] ($\Delta \varepsilon _r^{\prime } \le 1\%$ and $\Delta \varepsilon _r^{\prime \prime } \le 8\%$), validating the derived $G_{\text {obj}} (a)$ in (24) and $H_{\text {obj}} (b)$ in (25). To evaluate the effect of increased sample length on *a* computation using $G_{\text {obj}} (a)$ in (24) and $H_{\text {obj}} (b)$ in (25), they were re-drawn as shown in Fig. 4a,b for the tested sample with *W = 8.0* mm (all the remaining parameters are the same). It is seen from the results in Fig. 4a,b that there are more than one *a* and *b* pair producing minimum $G_{\text {obj}} (a)$ and $H_{\text {obj}} (b)$ when *W > λ /4*, meaning that $G_{\text {obj}} (a)$ in (24) and $H_{\text {obj}} (b)$ in (25) are effective for electrically thinner samples (*W < λ /4*) only for unique $\varepsilon _r$ determination. As a consequence, the maximum thickness $W_{\text {max}}$ that $G_{\text {obj}} (a)$ is applicable is limited by $W_{\text {max}} \le \lambda /4$ or $W_{\text {max}} \le \lambda _0/(4 a)$ where $\lambda _0$ is the free-space wavelength.

## Effect of sample misalignment

In our previous study^21^, sensitivity and uncertainty analyses were performed utilizing closed-form expressions and GUM-based uncertainty model^34^. Such analyses are important to evaluate the performance of a new extraction method. Although our new model in Fig. 1 is more general than that in Fig. 1 of our previous study^21^, as noted in “Introduction” and “Measurement results and discussion”, the fundamental objective functions ((23) in our present study and (18) in the study^21^) are identical. Therefore, sensitivity and uncertainty analyses performed in the study^21^ are still applicable for our new model in Fig. 1. However, not mentioned and examined in that study^21^ was the effect of sample misalignment on $\varepsilon _{r}$ evaluation. To fill in this gap, in the present study, sample misalignment effect was examined.

**Figure 5:**
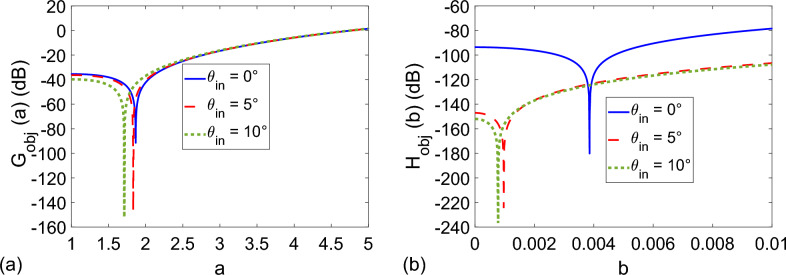
Dependencies of (**a**) $G_{\text {obj}} (a)$ versus *a* and (**b**) $H_{\text {obj}} (b)$ versus *b* of the tested sample (*W = 2.00* mm) under different incidence angle ($\theta _{\text {in}}$) values.

**Figure 6:**
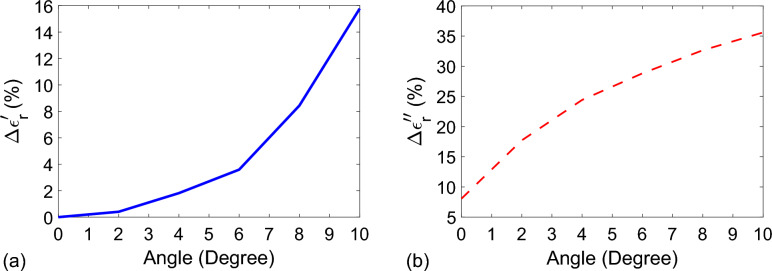
Percentage variations of (**a**) $\varepsilon _r^{\prime }$ and (**b**) $\varepsilon _r^{\prime \prime }$ versus incidence angles of the tested sample (*W = 2.00* mm).

To achieve our goal, 3D simulations using CST Microwave Studio were carried out. To realize a sample with a theoretically infinite transverse plane, unit cell boundary conditions were implemented over the transverse plane of the sample in the frequency domain by selecting the tetrahedral mesh-type (adaptive meshing) to perform the simulations precisely. Simulations were conducted at X-band (8.2 - 12.4 GHz) with 1001 frequency (default) points. Figure 5a,b illustrate, respectively, the dependencies of $G_{\text {obj}} (a)$ versus *a* and $H_{\text {obj}} (b)$ versus *b* for the tested sample (*W = 2.00* mm) under different incidence angle ($\theta _{\text {in}}$) values. It is seen from Fig. 5a,b that our proposed method requires precise positioning for an accurate *a* and *b* determination, since $G_{\text {obj}} (a)$ and $H_{\text {obj}} (b)$ are chiefly sensitive to $\theta _{\text {in}}$. Besides, Fig. 6a,b demonstrate, respectively, the percentage variations ($\Delta \varepsilon _r^{\prime }$ and $\Delta \varepsilon _r^{\prime \prime }$) of $\varepsilon _r^{\prime }$ and $\varepsilon _r^{\prime \prime }$ versus incidence angles. It is noted from the results in Fig. 6a,b that precise sample positioning is critical for an accurate $\varepsilon _r$ determination (especially for $\varepsilon _r^{\prime \prime }$ because the sample is a very low-loss sample).

## Measurement results and discussion

**Figure 7:**
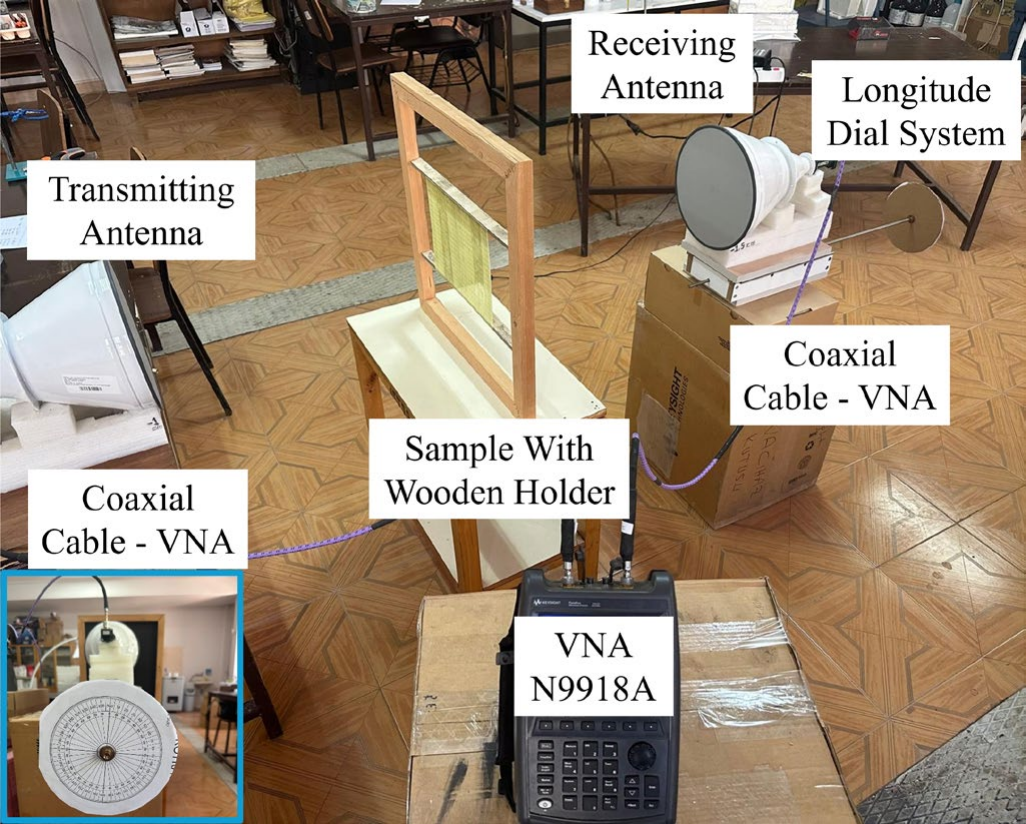
Measurement setup for validation of our proposed method.

Figure 7 presents a photo of the free-space measurement setup used to validate our proposed method. This setup includes a vector network analyzer (VNA), manufactured by the Keysight Technologies (Model: N9918A), to perform 2-port S-parameter measurements at X-band. Electromagnetic signals generated by the VNA are carried out by two coaxial lines to identical horn-lens antennas, manufactured by the Flann Microwave Instruments (Series 820). Our theoretical model assumes that the sample is positioned at the far-field (or far-zone) of the transmitting and receiving antennas. For an ordinary lens-free (lensless) antenna, the far-field location could be set to a distance at least the limit of $2 D^2/\lambda$ where *D* is the largest dimension of the antenna (the diagonal distance for rectangular antennas and diameter for circular antennas) and *λ* is the wavelength. As seen from this expression, this criterion can be safely met by considering the minimum frequency of the operating frequency band. Besides, free-space measurements can suffer from diffraction effects arising from sharp edges or corners of a sample and (spherical) wave spreading effects while wave is propagating. These two effects set a limit for the transverse dimensions of the analyzed sample. A a rule of thumb, transverse dimensions (sizes) of the sample should be selected to be greater than at least 3 times the footprint (or -10 dB beam diameter) of the quasi-Gaussian beam at the sample surface^6^. Since achieving the far-field approximation and suppressing the aforementioned effects in a free-space setup are partly difficult for a lens-free antenna, in our measurement, we utilized a horn-lens antenna for convenience. Then, samples were positioned approximately 40 cm away from both transmitting and receiving antennas to implement far-field criterion, after following the antenna manual and performing some preliminary measurements. Besides, it is observed that samples with at least transverse dimensions of 20cm*× 20*cm has sufficient accuracy^21^ to mitigate, if not totally eliminate, diffraction and wave spreading effects in our measurement setup.

Different from our previous study in^21^, here two different measurement scenarios were considered in our present study. The first scenario (NZR-1 case) assumes that the two coaxial lines are identical with a length of 100 cm. The second scenario (NZR-2 case) considers that the first coaxial line is the same as the previous case, but the length of the second coaxial line is doubled (two identical coaxial lines each of which has a length of 100 cm are cascaded sequentially). This means that while the first scenario is the scenario that was considered in our previous study^21^, the second scenario is a new one, which exemplifies the free-space model given in Fig. 1. Two different samples^21^ with diverse $\varepsilon _r^{\prime }$ and $\varepsilon _r^{\prime \prime }$ values were tested to assess the performance of the proposed method. These samples are the Rogers 4350B sample (*W ≈ 1.52* mm) and the FR4 sample (*W ≈ 1.60* mm) with their lengths measured by a high precision caliper with a resolution of 0.05 mm over 200 mm scale (from Mitutoyo with a dial reading).

A rectangular hollow wooden frame (around *40 × 40* cm$^2$) was utilized to firmly hold the samples to eliminate angle misalignment inline with the results in “Effect of sample misalignment”. Tested samples were sequentially positioned around the center of the frame. A longitudinal stage was used to precisely arrange the distance between either antenna and the sample surface (see the inset of Fig. 7). S-parameter measurements (total of 1001 frequency points linearly located at X-band) were iterated five times for each sample to examine repeatability of the extraction methods to be discussed shortly.

**Figure 8:**
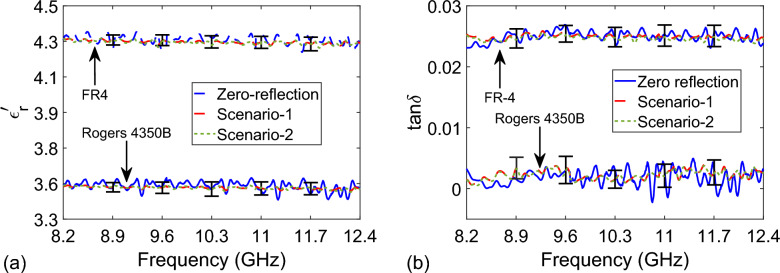
Measured (**a**) $\varepsilon _r^{\prime }$ and (**b**) tan*δ* of the tested Rogers 4350B and FR-4 samples by the proposed method for difference cases: i) Zero reflection ($S_{11}^{(a)} = 0$ and $S_{22}^{(a)} = 0$), ii) Scenario-1 involving $S_{11}^{(a)} \ne 0$ and $S_{22}^{(a)} \ne 0$ (two identical coaxial lines), and iii) Scenario-2 involving $S_{11}^{(a)} \ne 0$ and $S_{22}^{(a)} \ne 0$ (two non-identical coaxial lines).

Figure 8a,b show, respectively, the extracted $\varepsilon _r^{\prime }$ and $\varepsilon _r^{\prime \prime }$ of the tested Rogers 4350B and FR-4 samples under three different cases using averaged S-parameters of five independent measurements along with the rolling average method implemented for ten frequency points to eliminate residual parasitic signals^21^. These figures also indicate the percentage variations of $\varepsilon _r$ and tan*δ* at frequencies 8.9 GHz, 9.6 GHz, 10.3 GHz, 11.0 GHz, and 11.7 GHz for the proposed method. The first case (ZR case) assumes that the transmitting and reflecting antennas have the same matrices (*[T] = [R]*) with no internal reflections ($S_{11}^{(a)} = 0$ and $S_{22}^{(a)} = 0$). This case was taken into account in the studies^14,16,18^ and in the studies^19,20^ (with no time-gating procedure). The second case (NZR-1) exemplifies that the transmitting and reflecting antennas have the same matrices (*[T] = [R]*) with non-identical and non-zero reflections ($S_{11}^{(a)} \ne S_{22}^{(a)} \ne 0$), which has been considered in our recent study^21^. The final case (NZR-2), which is analyzed in the present study, models the most general case that the transmitting and reflecting antennas have different matrices (*[T] ≠ [R]*) involving non-identical and non-zero reflections and transmissions. The following points are observed from the results in Fig. 8a,b. First, extracted and reference values ($\varepsilon _r = 3.48(1 - j 0.0037)$^33^ and $\varepsilon _r = 4.3(1 - j 0.025)$^35^) of $\varepsilon _r^{\prime }$ and tan*δ* of the tested Rogers 4350B and FR-4 samples are in good agreement over the entire frequency band. Second, retrieved $\varepsilon _r^{\prime }$ and tan*δ* of both samples for the NZR-1 and NZR-2 cases have lower oscillatory behavior (ripples) than those for the zero reflection case (ZR case). Such an oscillatory behavior is chiefly associated with disparity at antenna regions and non-negligible reflections (ringing effect) between antennas and each sample. Third, this ringing effect produce tan*δ* values lower than zero (non-physical) at some frequencies (e.g., 10.72 GHz) for the tested Rogers 4350B sample for the zero reflection case (ZR case).

Inline with our recent study^21^, the performances of the aforementioned three different cases (ZR, NZR-1, and NZR-2) were examined using quantitative error estimates (using five independent measurements). Tables 2 and 3 give evaluated $\varepsilon _r$ and tan*δ* of the Rogers 4350B and FR-4 samples at *f = 8.2* GHz (the minimum frequency), 10.3 GHz (the mid-frequency), and 12.4 GHz (the maximum frequency) frequencies for all three cases after utilizing the GUM-uncertainty model analyzed in^21^ ($\Delta |F_{\text {obj}}| = 0.05$, $\Delta \theta _{\text {obj}} = 1^o$, and *Δ W = 0.01* mm). The following three main deductions can be inferred from the results in Tables 2 and 3. First, retrieved $\varepsilon _r$ has lower percentage variations than *δ* has for either sample mainly due to low-loss nature of tested samples and reduced accuracy of non-resonant methods for low-loss samples. Second, the NZR-1 and NZR-2 cases have comparatively lower $\varepsilon _r$ and tan*δ* percentage variations than the ZR case for both tested samples at all discrete frequencies. We think that this is the composite effects of disparity at antenna regions and non-negligible reflections (ringing effect) between antennas and each sample. Third, the NZR-1 and NZR-2 cases have close percentage variations for $\varepsilon _r$ and tan*δ* for both samples at all discrete frequencies. This is because NZR-1 and NZR-2 cases utilize the same objective function $F_{\text {obj}}$ in (23). Nonetheless, the free-space model in Fig. 1 is more general that those considered in the studies^16,19,21^, suitable for applications involving a free-space measurement setup having two different transmitting and receiving antenna regions (e.g., *[T] ≠ [R]*).

**Table 2:** Evaluated $\varepsilon _r$ and tan*δ* of the Rogers 4350B sample at some discrete frequencies (*f = 8.2* GHz, 10.3 GHz, and 12.4 GHz) for three different cases: i) ZR case^16,19^, ii) NZR-1 case^21^, and iii) NZR-2 (the model in Fig. 1 in the present study).

	Case	*f = 8.2* GHz	*f = 10.3* GHz	*f = 12.4* GHz
$\varepsilon _r^{\prime }$	ZR	$3.49 \mp 4.12\%$	$3.47 \mp 4.35\%$	$3.47 \mp 4.78\%$
	NZR-1	$3.48 \mp 3.47\%$	$3.47 \mp 3.12\%$	$3.47 \mp 3.45\%$
	NZR-2	$3.48 \mp 3.43\%$	$3.47 \mp 3.10\%$	$3.47 \mp 3.46\%$
tan*δ*	ZR	$0.0032 \mp 8.11\%$	$0.0030 \mp 8.78\%$	$0.0029 \mp 9.67\%$
	NZR-1	$0.0014 \mp 6.12\%$	$0.0015 \mp 5.89\%$	$0.0027 \mp 6.26\%$
	NZR-2	$0.0013 \mp 5.97\%$	$0.0015 \mp 5.56\%$	$0.0026 \mp 6.25\%$

**Table 3:** Evaluated $\varepsilon _r$ and tan*δ* of the FR-4 sample at some discrete frequencies (*f = 8.2* GHz, 10.3 GHz, and 12.4 GHz) for three different cases: i) ZR case^16,19^, ii) NZR-1 case^21^, and iii) NZR-2 (the model in Fig. 1 in the present study).

	Case	*f = 8.2* GHz	*f = 10.3* GHz	*f = 12.4* GHz
$\varepsilon _r^{\prime }$	ZR	$4.31 \mp 3.63\%$	$4.32 \mp 3.48\%$	$4.31 \mp 4.01\%$
	NZR-1	$4.31 \mp 2.84\%$	$4.30 \mp 2.75\%$	$4.29 \mp 2.64\%$
	NZR-2	$4.31 \mp 2.83\%$	$4.30 \mp 2.75\%$	$4.29 \mp 2.64\%$
tan*δ*	ZR	$0.023 \mp 8.35\%$	$0.024 \mp 8.41\%$	$0.023 \mp 8.95\%$
	NZR-1	$0.025 \mp 6.38\%$	$0.024 \mp 6.11\%$	$0.023 \mp 5.98\%$
	NZR-2	$0.025 \mp 6.35\%$	$0.024 \mp 6.13\%$	$0.023 \mp 5.78\%$

## Advantages and limitations of our method

A general free-space model considering the case that *[T] ≠ [R]* is examined in the present study. An objective function based on this model and specialized for electrically thinner samples is derived with no requirement of any formal calibration procedure such as the TRL method, unlike previous extraction methods in the literature^4–13,15,17,19,21,22^. Additionally, our extraction method uses both reflection and transmission measurements, providing more information at one frequency than other reflection-only methods^14,18,20^. Nonetheless, our method requires reflection measurements along with transmission measurements, which are not feasible for applications where the other side of the sample is not accessible. As the second drawback, the samples are assumed to be free-standing with no support by any sample holder. This means that for extremely thinner samples, the sample sagging^5^ plays a critical role for limiting the accuracy of the proposed method due to non-uniform sample surface pattern. As another limitation, precise sample thickness needs to be provided for improved $\varepsilon _r$ determination by the proposed method. As the final drawback of our method, angle misalignment lowers the accuracy of our method, as considered in “Effect of sample misalignment”. Thus, a proper sample alignment in reference with antenna apertures is a critical factor for accurate $\varepsilon _r$ determination by the proposed method.

On the other hand, it is instructive to discuss that the objective function $F_{\text {obj}}$ in (23) is valid for thinner (*W < λ /4*) or thicker (*W > λ /4*) dielectric samples with low-loss, medium-loss, or high-loss behavior, since there was not any assumption enforced over it in its derivation procedure. Nevertheless, the objective functions $G_{\text {obj}}$ in (24) and $H_{\text {obj}}$ in (25) are strictly applicable for thinner low-loss dielectric samples only because $\text {sinh} (b \phi _0) \approx b \phi _0$ and $\text {cosh} (b \phi _0) \approx 1.0$ assumptions were exercised in their derivations. In other words, if a thicker low-loss (medium-loss or high-loss) dielectric sample is being characterized, then $F_{\text {obj}}$ could be directly utilized to determine $\varepsilon _r$. However, it is noteworthy that $F_{\text {obj}}$ then necessitates a good initial guess $\varepsilon _r$ for a thicker low-loss (medium-loss or high-loss) dielectric sample, because $\text {cos} [ (1 - \sqrt{\varepsilon _r} ) k_0 W]$ and $\text {cos} [ (1 + \sqrt{\varepsilon _r} ) k_0 W]$ terms are multi-valued terms. Finally, it is noted that all the objective functions ($F_{\text {obj}}$, $G_{\text {obj}}$, and $H_{\text {obj}}$) derived in this present study assume that the sample under examination is a dielectric sample. The terms *Γ* and *T* in (21) could be generalized to reflect magnetic behavior of a general (magnetic) sample:

27 $$\begin{aligned} \Gamma = \frac{ \sqrt{ \frac{\mu _r}{\varepsilon _r} } - 1 }{ \sqrt{ \frac{\mu _r}{\varepsilon _r} } + 1 } \end{aligned}$$

28 $$\begin{aligned} T = e^{ -j k_0 \sqrt{ \mu _r \varepsilon _r} W }, \end{aligned}$$

where $\mu _r$ is the magnetic permeability of the sample. Since, then, there is one complex (real and imaginary parts) measurement data but two complex unknowns ($\varepsilon _r$ and $\mu _r$), the system turns into an underdetermined system, meaning there are fewer equations (constraints) than unknowns (variables) with infinitely many solutions rather than a single unique solution. For this reason, we constrained our analysis to dielectric samples only in the present study. In future, our goal is to determine unique solution for both $\varepsilon _r$ and $\mu _r$ considering an additional measurement or additional measurements.

## Conclusion

A theoretical model generalizing the configuration of a free-space measurement environment is considered in this study. Distinct from previous free-space models, the proposed model takes into account of different error network models for transmitting and receiving antennas with non-zero and non-identical reflection and transmission properties. Such a model applicable for any frequency or over the entire frequency band examines different antenna characteristics involving impedance mismatches and ringing effects between antennas and the sample. An objective function is derived for $\varepsilon _r$ retrieval of low-loss and electrically thin samples based upon this generalized model, with the advantages of non-dependence on error network elements and reference plane transformation factors. From the analysis of angle misalignment effect, it is noted that the proposed method requires precise sample alignment to improve the accuracy of $\varepsilon _r$ evaluation. Free-space measurements of $\varepsilon _r$ of two low-loss and electrically thin samples (the Rogers 4350B sample with *W ≈ 1.52* mm and the FR-4 sample with *W ≈ 1.60* mm) are performed to validate and evaluate the performance of the proposed method.

## Data Availability

The datasets used and analyzed during the current study are available from the corresponding author (U.C.H) upon reasonable request.
